# Supplementary material for: Metabolic Signatures of 10 Processed and Non-processed Meat Products after In Vitro Digestion
Source: Metabolites. 2020 Jul 3;10(7):272. doi: 10.3390/metabo10070272 (PMC7408382; doi:10.3390/metabo10070272)
Supplement: Supplementary file 1 [file metabolites-10-00272-s001.zip › metabolites-830081-supplementary-re2/Supplement Meat digest.pdf]

Online supporting material

In vitro digests of meat products reveal specific metabolic signature of processed meat products in an untargeted metabolomics analysis

Roland Wedekind [1], Pekka Keski-Rahkonen[1], Nivonirina Robinot[1], Frederic Mercier[2], Erwan Engel[2], Inge Huybrechts[1] and Augustin Scalbert[1,*]

[1] Nutrition and Metabolism Section, International Agency for Research on Cancer, Lyon, France
[2] Micro-contaminants, Aroma and Separation Sciences (MASS) Group, National Research Institute for Agriculture, Food and Environment (INRAE) UR370 QuaPA, Saint-Genès-Champanelle, France
* Correspondence: scalberta@iarc.fr

TABLE S1: See excel sheet
TABLE S2: Metabolic features with significantly higher levels in processed meat digests compared to non-processed pork and beef digests. 2
TABLE S3 : Features significantly elevated in processed meat digests compared to non-processed meat products and found in pepper extracts. 12
TABLE S4 : Details on the recursive feature finding used to create the feature table from the HRMS raw data. 14

FIGURE S1 : Annotation evidence for Pellironine in fried sausage digest 15
FIGURE S2 : Annotation evidence for Piperyline 16
FIGURE S3 : Annotation evidence for Piperanine 17
FIGURE S4 : Annotation evidence for Piperine 18
FIGURE S5 : Annotation evidence for Piperettine 19
FIGURE S6 : Annotation evidence for Piperdardine 20
FIGURE S7 : Annotation evidence for pepper compound 2 21
FIGURE S8 : Annotation evidence for pepper compound 3 22
FIGURE S9 : Annotation evidence for pepper compound 4 23
FIGURE S10 : Annotation evidence for pepper compound 5 24
FIGURE S11 : Annotation evidence for pepper compound 6 25
FIGURE S12 : Annotation evidence for pepper compound 7 26
FIGURE S13 : Annotation evidence for pepper compound 8 27
FIGURE S14 : Annotation evidence for pipercide 28
FIGURE S15 : Annotation evidence for phenylalanylisoleucine. 29
FIGURE S16 : Annotation evidence for γ-aminobutyric acid 30
FIGURE S17 : Annotation evidence for histamine in fermented sausage digest 31
FIGURE S18: Annotation evidence for tyramine 32
FIGURE S19 : Annotation evidence for tryptamine in fermented sausage digest 33

References 34

TABLE S2: Metabolic features with significantly higher levels in processed meat digests compared to non-processed pork and beef digests. A Welch's t-test revealed 178 metabolic features that were significantly (FDR-adjusted p-value <0.05) and with at least 2-fold increase in processed meat products (n = 63) compared to non-processed pork and beef (n = 36).

m/z	RT	FDR-adjusted p-value	Fold-change[a]	Cluster[b]	Adduct	HMDB ID[c]	Annotation	Annotation level of confidence[d]
359.2448	7.13	3.71E-06	2.31	1				
523.3828	7.14	2.35E-06	2.77	1				
241.1416	5.48	0.000339	2.10	1				
265.1778	6.66	0.00011	2.24	1				
570.8279	4.92	2.35E-06	2.01	1				
669.3921	3.93	2.33E-06	2.19	1				
581.317	4.92	9.77E-07	2.26	1				
631.4697	9.75	3.36E-06	2.19	1				
652.5865	9.75	6.41E-06	2.22	1				
685.5394	7.87	0.000228	2.04	1				
522.8854	7.13	9.28E-05	2.04	1				
575.502	9.75	1.01E-05	2.27	1				
531.4017	8.15	0.000601	2.03	1	[M+Na]+	HMDB0007038	**Unknown 1:** Possibly diglyceride	4

347.2192	6.69	0.000182	2.14	1				
353.2662	7.26	0.007331	2.21	1	[M+Na]+	HMDB0011533	**Unknown 2**: Possibly monoglyceride	4
302.1623	5.92	0.002954	2.34	1				
325.2363	6.18	0.002154	2.03	1	[M+Na]+	HMDB0011530	**Unknown 3:** Possibly monoglyceride	4
793.5126	7.04	0.000432	2.12	1				
811.4872	7.04	0.002099	2.06	1				
337.272	9.75	0.000136	2.72	1				
263.2366	7.78	0.000116	2.77	1				
608.524	9.30	0.013896	2.17	1				
364.254	6.92	0.001657	2.57	1				
361.1687	6.76	0.002712	2.19	1				
271.2271	6.48	0.000799	3.45	1				
312.2536	6.69	0.018251	2.09	1				
615.4575	6.75	0.005894	2.18	1				
317.1556	6.12	0.016059	2.08	1				
409.2028	7.78	0.004203	2.43	1				
315.1643	6.12	0.006405	2.12	1				
421.2532	7.21	0.002053	2.93	1				
353.2669	7.68	0.019551	2.30	1	[M+Na]+	HMDB0011533	**Unknown 4** : Possible monoglyceride	4

369.2406	7.18	0.01056	2.07	1				
279.1712	3.36	0.015885	3.13	2	$[M+H]^+$	HMDB0028998	**Phe-Ile dipeptide**	2
245.1381	4.32	0.019551	2.34	2				
523.383	7.17	0.007815	3.80	2				
524.3847	7.17	1.65E-05	501.94	2				
332.2184	3.63	0.02459	3.50	2				
360.2896	6.78	0.01454	4.36	2				
145.0496	0.86	3.67E-05	415.11	2				
244.9731	1.86	0.00029	14.42	2				
285.1313	4.17	0.000112	327.15	2				
261.0284	1.86	0.000513	306.60	2				
336.1922	3.22	0.000807	295.48	2				
127.0383	3.03	0.001373	262.82	2				
167.0312	1.53	0.004018	216.55	2				
379.2782	7.04	0.000233	2.89	3				
280.2356	6.72	0.010854	2.06	3				
365.2294	6.18	0.000737	2.56	3				
260.1949	0.85	0.000147	3.53	3				
581.8191	4.92	2.35E-06	2.48	3				

615.349	3.76	8.40E-05	2.70	3				
213.1105	4.45	2.94E-08	7.23	3				
172.0058	3.07	6.93E-06	2.69	3				
130.0323	2.42	8.12E-05	2.71	3				
176.0364	0.89	1.73E-06	3.35	3				
539.319	6.91	5.74E-05	4.34	3				
637.4798	8.99	7.39E-05	3.10	3				
632.5247	8.99	2.17E-06	3.93	3				
217.0851	5.22	5.29E-05	5.03	3				
402.2352	3.30	1.86E-05	5.67	3				
754.4483	4.62	7.64E-07	9.90	4				
308.1262	6.01	3.66E-05	3.80	4	[M+Na]+	HMDB0029377	**Piperine**	1
393.3841	7.34	7.94E-05	2.52	4				
345.2181	5.99	9.03E-08	8.61	4				
272.1287	5.74	7.35E-05	3.47	4	$[M+H]^+$	HMDB0029374	**Piperyline**	2
312.1597	6.29	6.85E-07	10.83	4	$[M+H]^+$	HMDB0034371	**Piperettine**	2
288.1601	5.93	7.99E-08	13.03	4	$[M+H]^+$	HMDB0033874	**Piperanine**	2
340.1909	6.57	0.000624	3.09	4	$[M+H]^+$	HMDB0040811	**Dehydropipernonaline (Pepper compound 5)**	3
344.2236	6.78	4.43E-06	7.17	4	$[M+H]^+$	HMDB0030340	**Piperolein B (Pepper compound 7)**	3

403.2953	6.78	6.71E-09	681.21	4				
347.2317	5.93	2.29E-08	29.92	4				
224.2012	6.36	4.79E-07	12.10	4	$[M+H]^+$	HMDB0030951	**Pellitorine**	1
722.3292	6.01	1.50E-08	628.89	4				
700.3479	6.02	1.55E-08	601.29	4				
314.1751	6.28	1.61E-07	23.66	4				
371.2331	6.28	3.46E-08	686.62	4			**Compound in pepper**	3
331.2016	5.74	3.46E-08	606.90	4				
316.192	6.41	1.52E-07	586.71	4	$[M+H]^+$	HMDB0030185	**(E)-Piperolein A (Pepper compound 1)**	3
310.1422	5.93	1.58E-07	554.04	4	[M+Na]+	HMDB0033874	**Piperanine, also as [M+H]+**	2
755.4229	5.98	6.85E-07	23.03	4				
334.1411	6.28	2.08E-06	20.10	4	[M+Na]+	HMDB0034371	**Piperettine**	2
285.1363	5.99	6.47E-07	524.09	4	[M+Na]+		Comp from pepper	3
314.1394	4.46	7.49E-08	607.34	4	$[M+H]^+$	PubChem CID : 10086948	**Piperdardine**	3
362.3409	7.60	3.69E-07	531.77	4	$[M+H]^+$	HMDB0032032	2,4,14-Eicosatrienoic acid isobutylamide	3
421.4146	7.60	2.32E-07	23.64	4				
406.2351	6.97	0.001903	2.35	4	[M+Na]+	HMDB0030186	(E,E,E)-Sylvatine	3
593.2622	5.98	5.65E-05	4.37	4	[M+Na]+	HMDB0036360	Pipercyclobutanamide A	3
401.2776	6.67	0.001417	3.91	4				

274.1444	5.79	5.35E-06	443.90	4	$[M+H]^+$	HMDB0030187	Pepper compound	4
330.2072	6.62	1.26E-06	494.56	4	$[M+H]^+$	HMDB0039808	**Pipertipine (Pepper compound 4)**	3
366.2044	6.78	2.54E-06	472.13	4	[M+Na]+	HMDB0030340	**Piperolein B (Pepper compound 7)**	3
399.2626	6.56	4.70E-06	454.50	4				
326.1768	6.40	4.97E-06	437.90	4	$[M+H]^+$	HMDB0038644	**(2E,4E,8E)-Piperamide-C9:3 (Pepper compound 2)**	3
328.1913	6.38	2.56E-06	442.84	4	$[M+H]^+$	HMDB0038645	**(2E,8E)-Piperamide-C9:2 (Pepper compound 3)**	3
356.222	6.70	9.28E-06	421.51	4	$[M+H]^+$	HMDB0033449	**Pipercide**	2
389.2802	6.61	1.71E-05	405.30	4				
443.3264	6.97	1.78E-05	402.80	4				
342.2088	6.67	0.000208	11.43	4	$[M+H]^+$	HMDB0030339	**Pipernonaline (Pepper compound 6)**	2
337.2684	7.68	0.00142	2.64	5				
436.285	7.25	0.015124	2.53	5				
328.25	6.47	0.019999	2.95	5				
185.0753	3.45	0.000652	14.15	5				
362.3153	6.74	0.023118	7.40	5				
311.2942	7.23	0.022502	2.45	5				
651.424	6.76	0.000425	2.93	5				
193.1585	6.75	0.002837	2.72	5				
225.1845	6.75	0.003458	3.54	5				

179.1432	6.75	0.00031	3.39	5				
171.1382	6.75	0.001009	3.96	5				
341.2684	7.14	0.001067	3.51	5				
617.5135	9.38	0.003503	3.14	5	$[M+H]^+$	HMDB0007112	**Unknown 5** : Possibly diglyceride	4
676.5864	9.38	0.033529	2.20	5				
693.5442	7.67	0.002254	4.21	5	$[M+H]^+$	HMDB0007776	**Unknown 6:** Possibly diglyceride	4
721.047	7.04	0.031479	2.56	5				
869.5439	7.03	0.03205	2.80	5				
273.2595	7.27	0.035203	2.80	5				
391.2467	6.45	0.021811	3.39	5				
655.4702	9.38	0.000152	7.60	5	[M+H]+	HMDB0112171	**Unknown 7 :** Possibly lipid	4
351.2534	6.44	0.00908	7.40	5	[M+H]+	HMDB0011541	**Unknown 8**: Possibly monoglyceride	4
291.1935	6.52	9.83E-05	402.30	5				
269.2103	6.52	0.00286	8.77	5				
635.4554	6.75	0.000198	316.28	5				
619.4827	6.76	0.000194	310.22	5				
384.1969	6.76	0.033007	2.43	5				
231.1	5.67	0.00119	12.83	5				
175.0271	1.62	0.021831	2.93	5				

654.4484	6.89	0.014143	2.67	5				
124.0394	1.08	0.002177	4.04	5	[M+H]+	HMDB0001488	nicotinic acid	3
350.1542	6.61	0.001745	3.92	5				
266.9938	3.85	0.001026	7.12	5				
189.0501	3.85	0.001118	12.42	5				
199.1694	6.89	0.006623	195.35	5				
274.0747	3.26	0.036974	4.38	5				
263.2369	6.26	0.00427	211.63	5				
261.0949	0.76	0.007683	175.05	5				
348.2149	6.92	1.56E-13	35.35	6				
343.2605	6.92	3.68E-08	617.52	6				
113.0597	2.76	0.002122	2.70	6				
239.0234	4.01	0.002993	2.46	6				
254.9955	4.01	3.42E-05	3.95	6				
185.0421	0.74	7.24E-10	706.89	6				
127.0388	2.65	9.68E-06	455.62	6				
191.0686	4.57	1.88E-07	643.75	6	[M+Na]+	HMDB0029680	**4-methylsyringol**	1
197.0089	2.77	7.46E-08	574.52	6				
183.1018	5.08	0.000111	8.28	6	[M+H]+	HMDB0033394	**4-ethylsyringol**	1

169.086	4.57	5.34E-06	23.07	6	[M+H]+	HMDB0029680	**4-methylsyringol**	1
205.0841	5.08	3.20E-06	513.53	6	[M+Na]+	HMDB0033394	**4-ethylsyringol**	1
257.061	4.01	2.71E-06	447.57	6				
269.0107	4.57	5.43E-06	408.09	6				
190.0607	4.57	5.57E-06	409.16	6				
168.0782	4.57	5.70E-06	408.34	6				
268.0026	4.57	1.02E-05	404.13	6				
253.0391	4.57	1.85E-05	395.38	6				
211.0959	3.60	0.013716	2.97	6				
183.0642	3.63	0.017322	3.09	6				
409.2559	6.12	0.000492	260.44	6				
537.3002	6.73	0.000477	268.54	6				
397.3824	8.19	0.001554	175.01	6				
217.0846	5.33	0.001599	282.41	6	[M+Na]+	HMDB0037271	**4-allylsyringol**	1
219.1001	5.54	0.025749	7.05	6	[M+Na]+	HMDB0036226	**4-propylsyringol**	1
562.2922	3.70	0.018079	5.37	6				
627.3506	4.23	0.035012	7.22	6				
445.245	4.70	0.007605	221.20	6				
345.2213	4.18	0.007582	220.80	6				

445.7474	4.70	0.021726	10.58	6				
456.2355	4.70	0.043219	7.68	6				
472.2045	4.69	0.02006	159.52	6				
607.3806	3.63	0.012317	185.67	6				
195.102	5.32	0.019201	178.91	6	[M+H]+	HMDB0037271	**4-allylsyringol**	1
197.0813	3.84	0.049335	4.16	6				
194.0948	5.33	0.005164	197.66	6				
479.2973	3.24	0.006717	194.73	6				
271.0771	4.57	0.011452	163.49	6				
197.1173	5.54	0.020426	141.68	6	[M+H]+	HMDB0036226	**4-propylsyringol, [M+Na] above**	1
464.2185	4.70	0.032136	142.30	6				
216.0774	5.33	0.032155	138.03	6				
327.2068	3.64	0.032156	132.57	6				

Abbreviations: RT, Retention time; ppm: parts per million; [a] mean intensity of metabolite in processed meat products divided by mean intensity in non-processed meat products, [b] clusters of metabolites in figure 2, [c] identifier of the Human Metabolome Database (www.hmdb.ca), [d] level of confidence as described in Sumner et al. [1]

TABLE S3 : Features significantly elevated in processed meat digests compared to non-processed meat products and found in pepper extracts. Pepper origin of all compounds is confirmed by comparison of MS/MS spectra with corresponding compounds in pepper extracts. Some could be annotated based on comparison with chemical standards or comparison of their MS/MS spectra with those curated in public databases. References are found at the end of the Supplement.

m/z	RT[a] (min)	Adjusted p-value[b]	Adduct	Mass accuracy [c] (ppm)	HMDB ID/ PubChem CID	Annotation (in brackets annotation in Figure 2)	Level of confidence[d]	Detection (Pepper type)	Reference[e]
224.2012	6.34	4.79E-07	H	1	HMDB0030951	Pellitorine	1	A[f]	[2]
272.1287	5.74	7.35E-05	H	2	HMDB0029374	Piperyline	2	A	[3,4]
288.1601	5.93	7.99E-08	H	2	HMDB0033874	Piperanine	2	A	[3–5]
308.1262	6.01	3.66E-05	Na	2	HMDB0029377	Piperine	1	A	
312.1597	6.29	6.85E-07	H	1	HMDB0034371	Piperettine	2	A	[3]
314.1751	6.28	1.61E-07	H	0	PubChem CID 10086948	Piperdardine	2	A	[3]
316.192	6.41	1.52E-07	H	4	HMDB0030185	(E)-Piperolein A (Pepper compound 1)	3	A	
326.1768	6.40	4.97E-06	H	5	HMDB0038644	(2E,4E,8E)-Piperamide-C9:3 (Pepper compound 2)	3	A	
328.1913	6.38	2.56E-06	H	2	HMDB0038645	(2E,8E)-Piperamide-C9:2 (Pepper compound 3)	3	A	
330.2072	6.62	1.26E-06	H	2	HMDB0039808	Pipertipine OR retrofractamide C OR 8E-piperamideC9:1 (Pepper compound 4)	3	B, G	
340.1909	6.57	0.000624	H	0	HMDB0040811	Dehydropipernonaline (Pepper compound 5)	3	A	
342.2088	6.67	0.000208	H	7	HMDB0030339	Pipernonaline OR Retrofractamide D (Pepper compound 6)	3	A	
344.2236	6.78	4.43E-06	H	5	HMDB0030340	Piperolein B (Pepper compound 7)	3	A	
356.222	6.70	9.28E-06	H	4	HMDB0033449	Pipercide	2	A	[4]

[a] RT, Retention time; [b] FDR-adjusted p-value of a Welch's t-test comparing metabolite intensities in processed meat digests to non-processed meat digests; [c] accuracy of measured mass compared to the theoretical mass of the annotation; [d] annotation level of confidence: Identity confirmed by comparison of RT and MS/MS fragmentation pattern with chemical standard (level 1), Annotation based on match of RT and MS/MS fragmentation pattern with compound present in black pepper extract AND matching MSMS fragmentation pattern with literature spectra (level 2), Annotation based on match of RT and MS/MS fragmentation pattern

with compound present in black pepper extract (level 3); [e] literature references for MS/MS spectra of pepper compounds: see last page of supplement [f] Detection in extracts of ground pepper (n = 6): A, all (black, white, green); B, G (black, green)

TABLE S4 : Details on the recursive feature finding used to create the feature table from the HRMS raw data. All steps were carried out using the Agilent MassHunter software package (Mass Profiler Professional version B14.9.1, Qualitative Analysis version B06.00 and DA Reprocessor version B.05.00; Agilent technologies).

**First step: Molecular feature extraction**	
Mass range	50-1000 m/z
Peak height threshold	1500 counts
Ions	Limited to [M+H]+
Isotope grouping	Common organic molecules (no halogens), single-charged ions
Alignment	15 ppm, +/- 0.1 min
**Second step: Recursive analysis**	
Creation of targets	features detected in at least 3 samples
Chromatogram extraction	+/- 25 ppm and +/- 1.0 min around target
Alignment of features	15 ppm, +/- 0.1 min

FIGURE S1 : Annotation evidence for Pellironine in fried sausage digest, comparison with compound in black pepper extract.

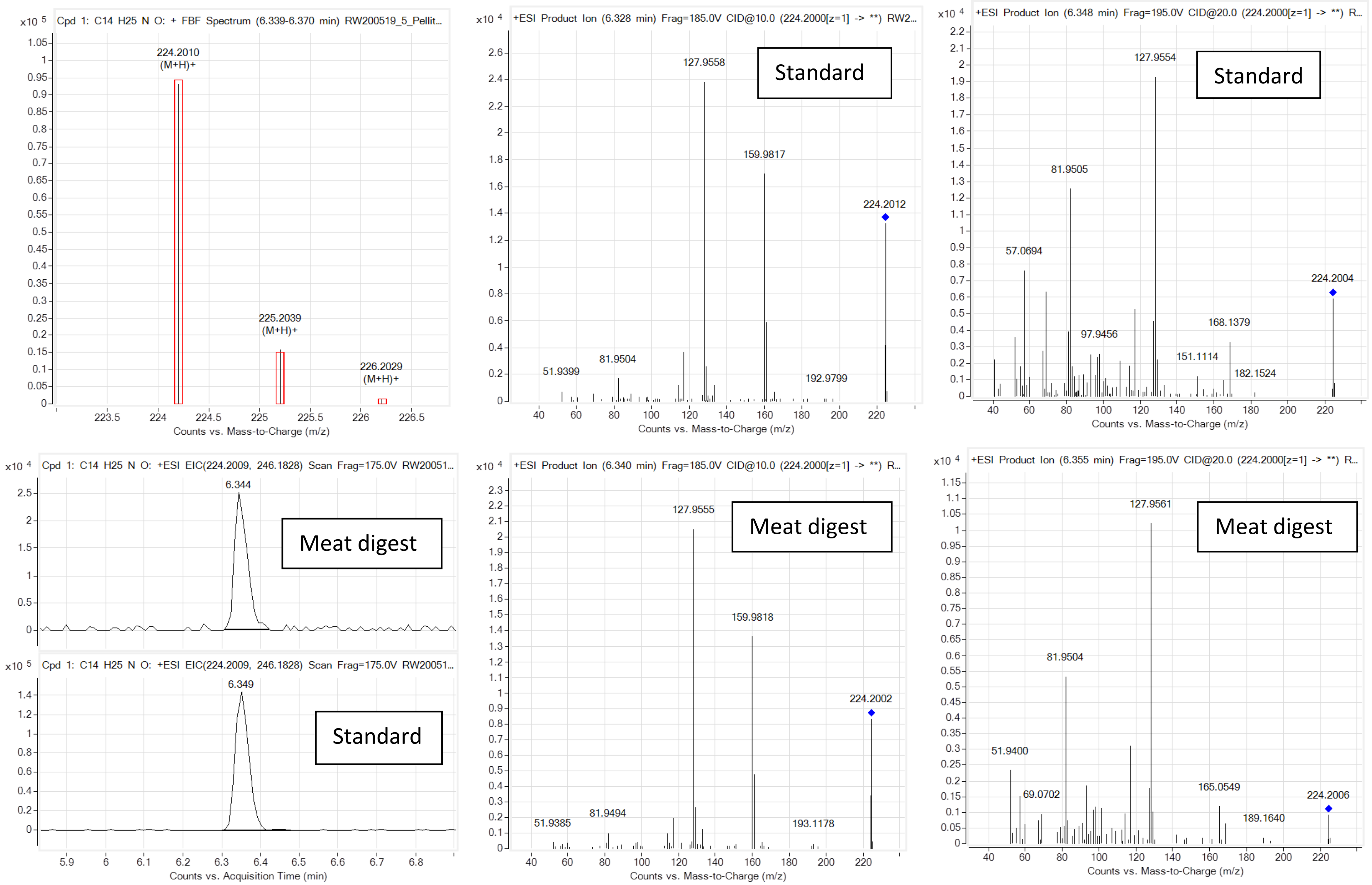

Online supporting material

FIGURE S2 : Annotation evidence for Piperyline in fried sausage digest, comparison with compound in black pepper extract.

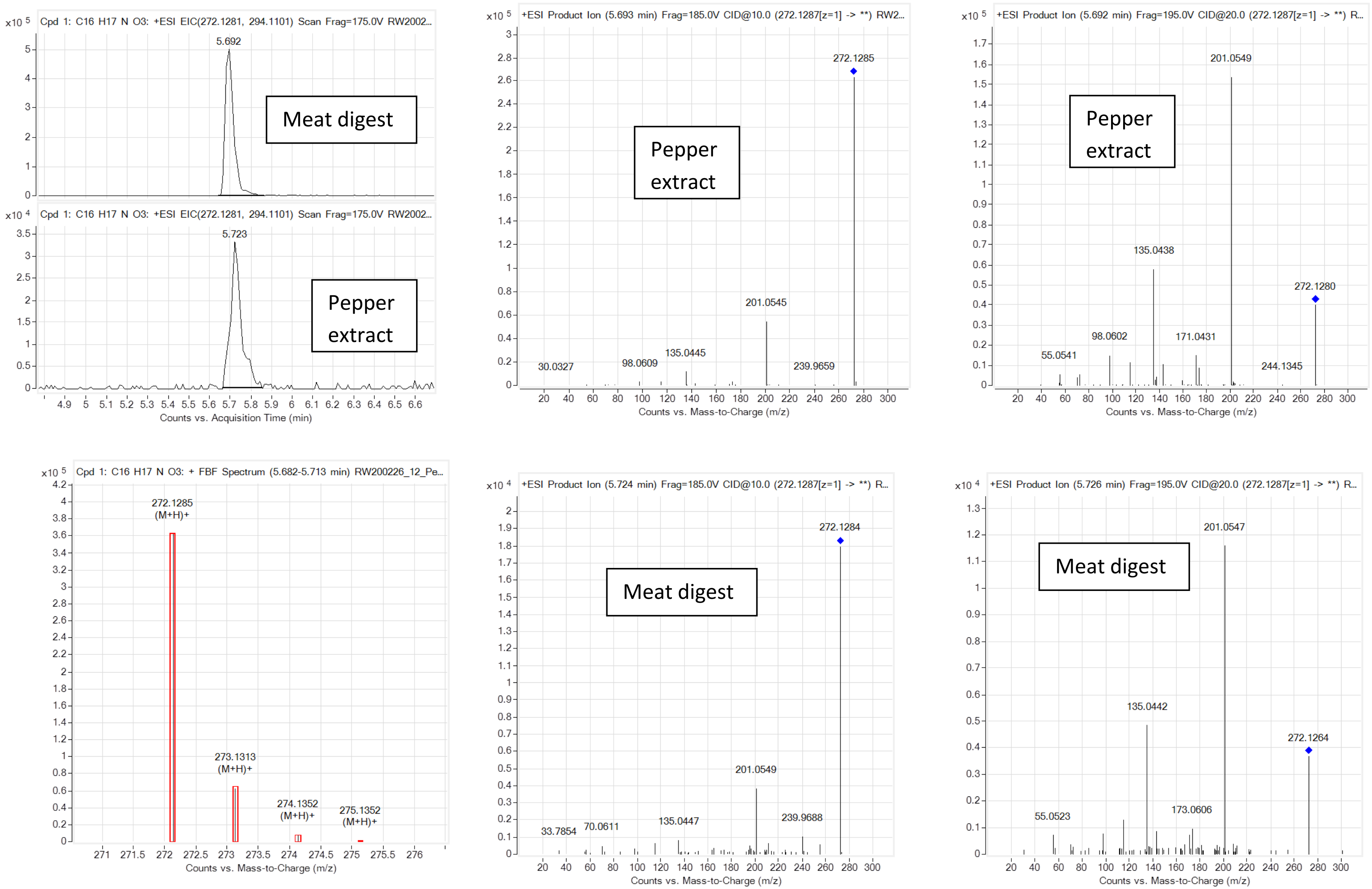

FIGURE S3 : Annotation evidence for Piperanine in fried sausage digest, comparison with compound in black pepper extract.

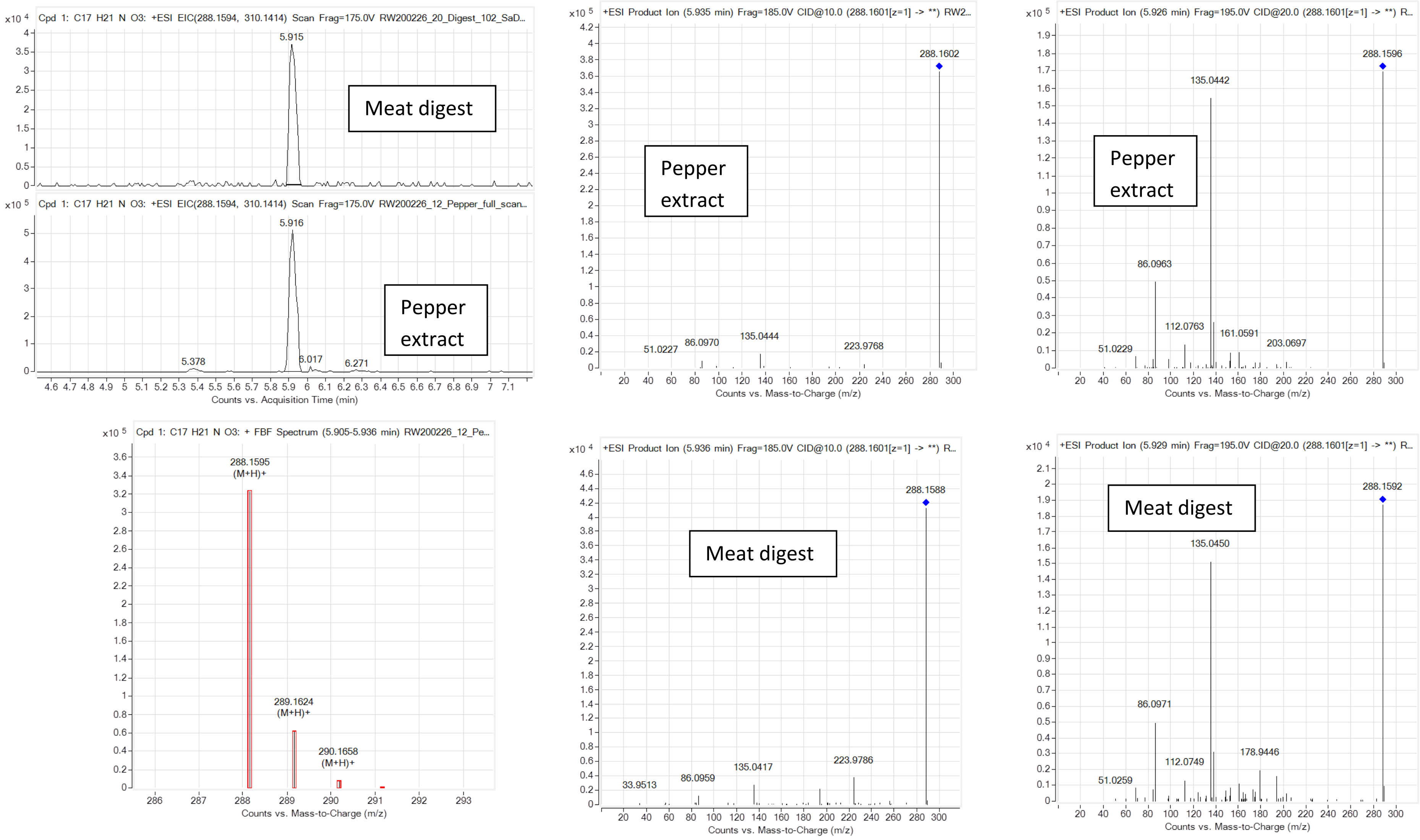

FIGURE S4 : Annotation evidence for Piperine in fried sausage digest, comparison with chemical standard and compound in black pepper extract.

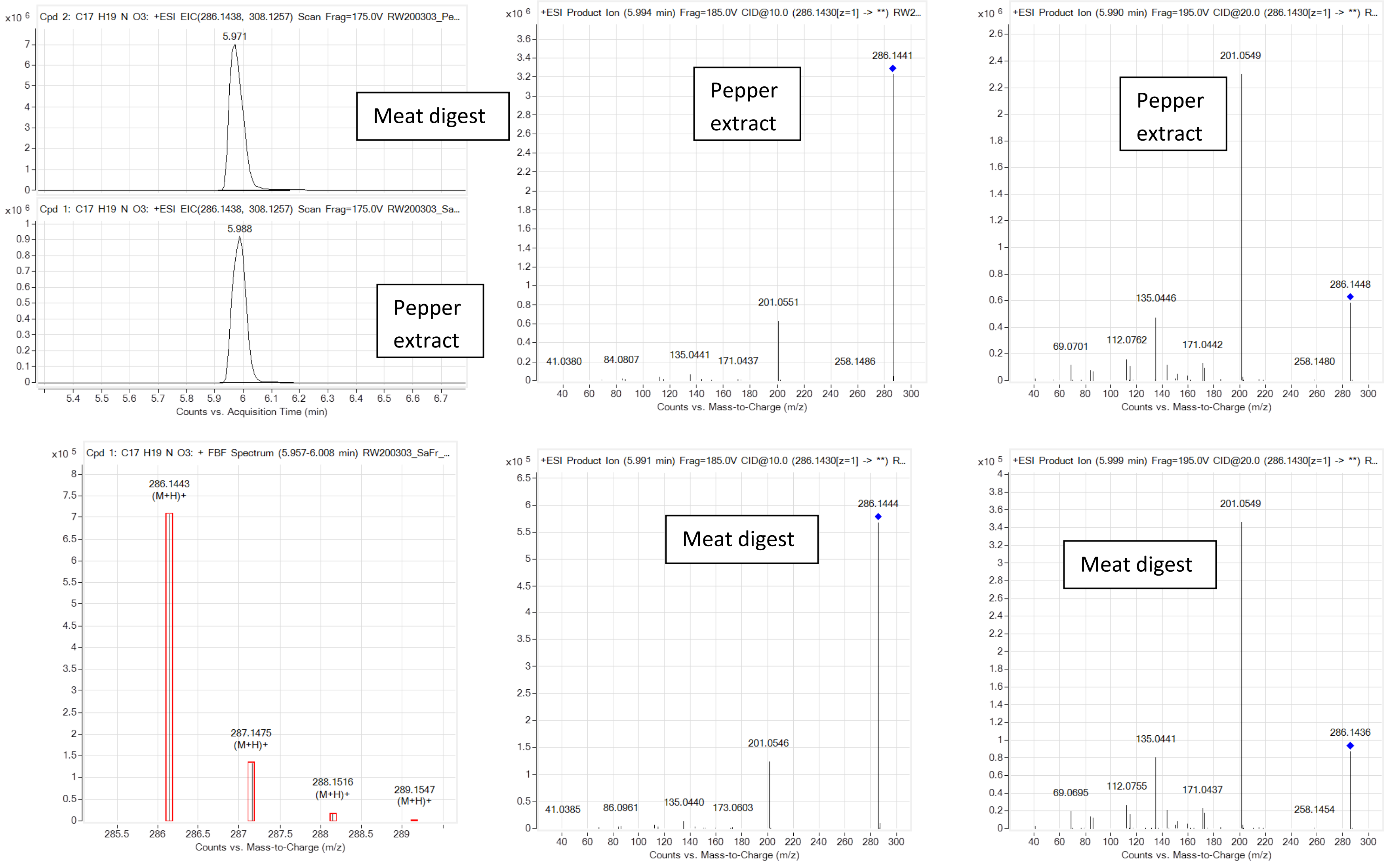

FIGURE S5 : Annotation evidence for Piperettine in fried sausage digest, comparison with compound in black pepper extract.

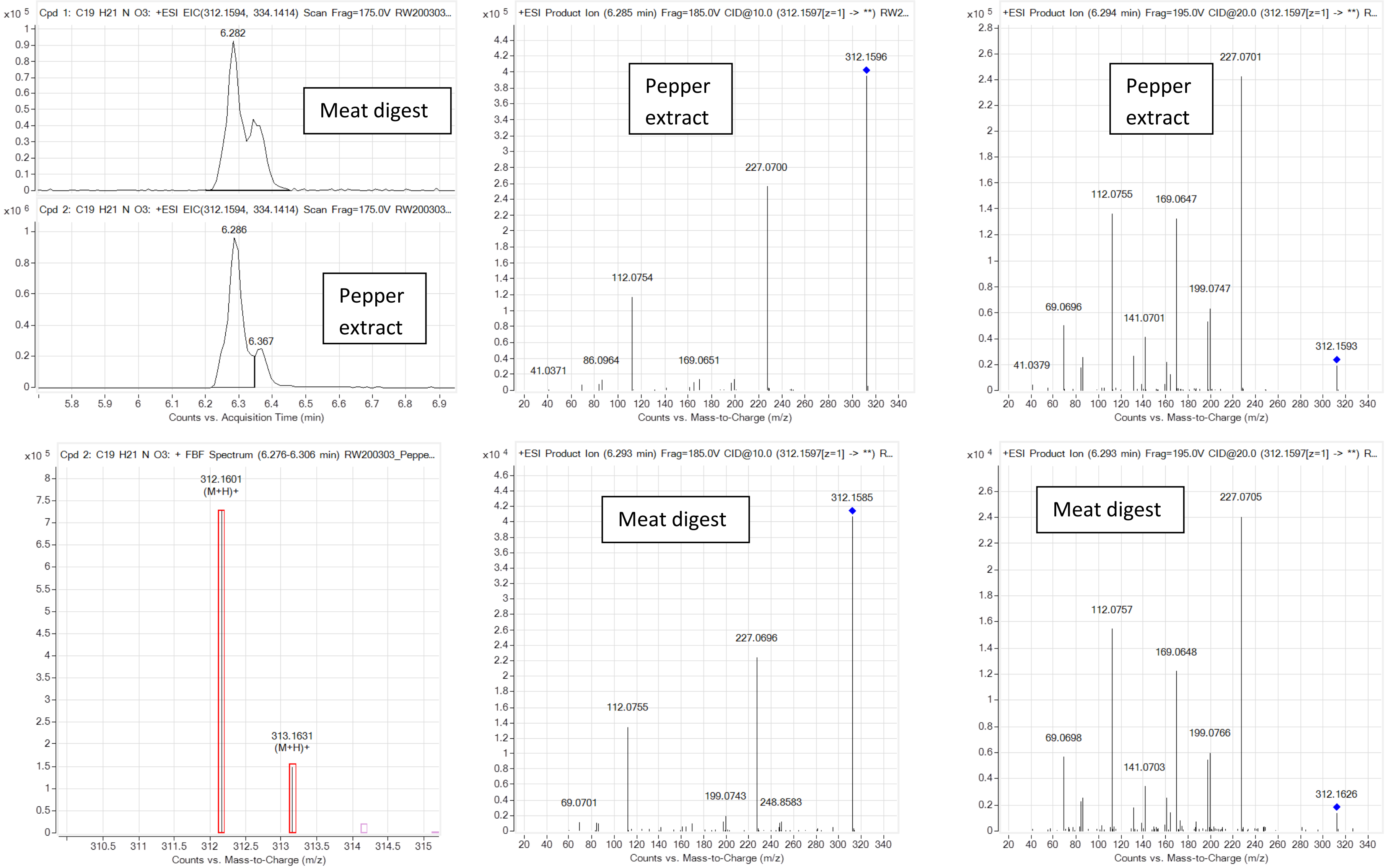

FIGURE S6 : Annotation evidence for Piperdardine in fried sausage digest, comparison with compound in black pepper extract.

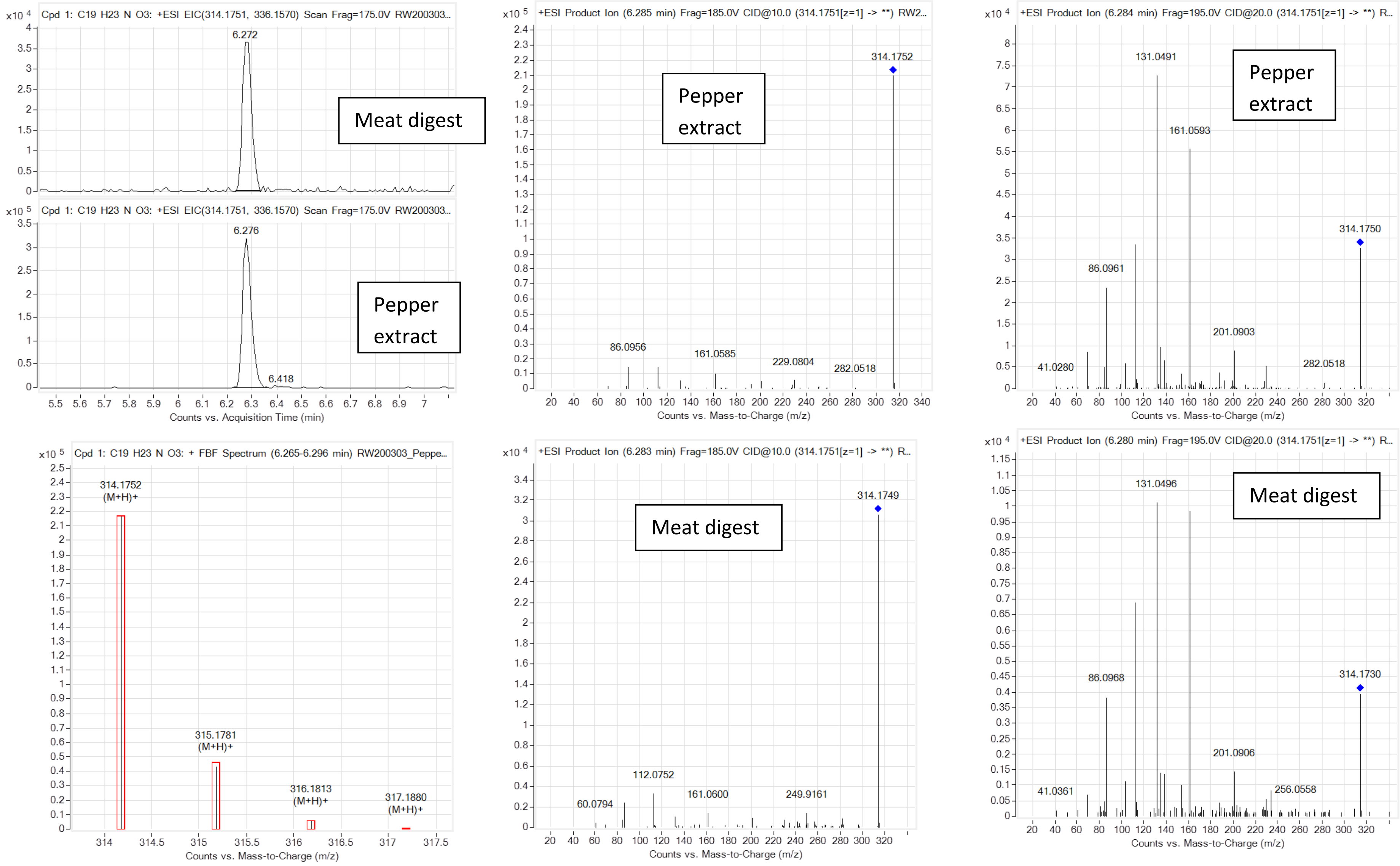

Online supporting material

FIGURE S7 : Annotation evidence for pepper compound 2 in fried sausage digest, comparison with compound in black pepper extract.

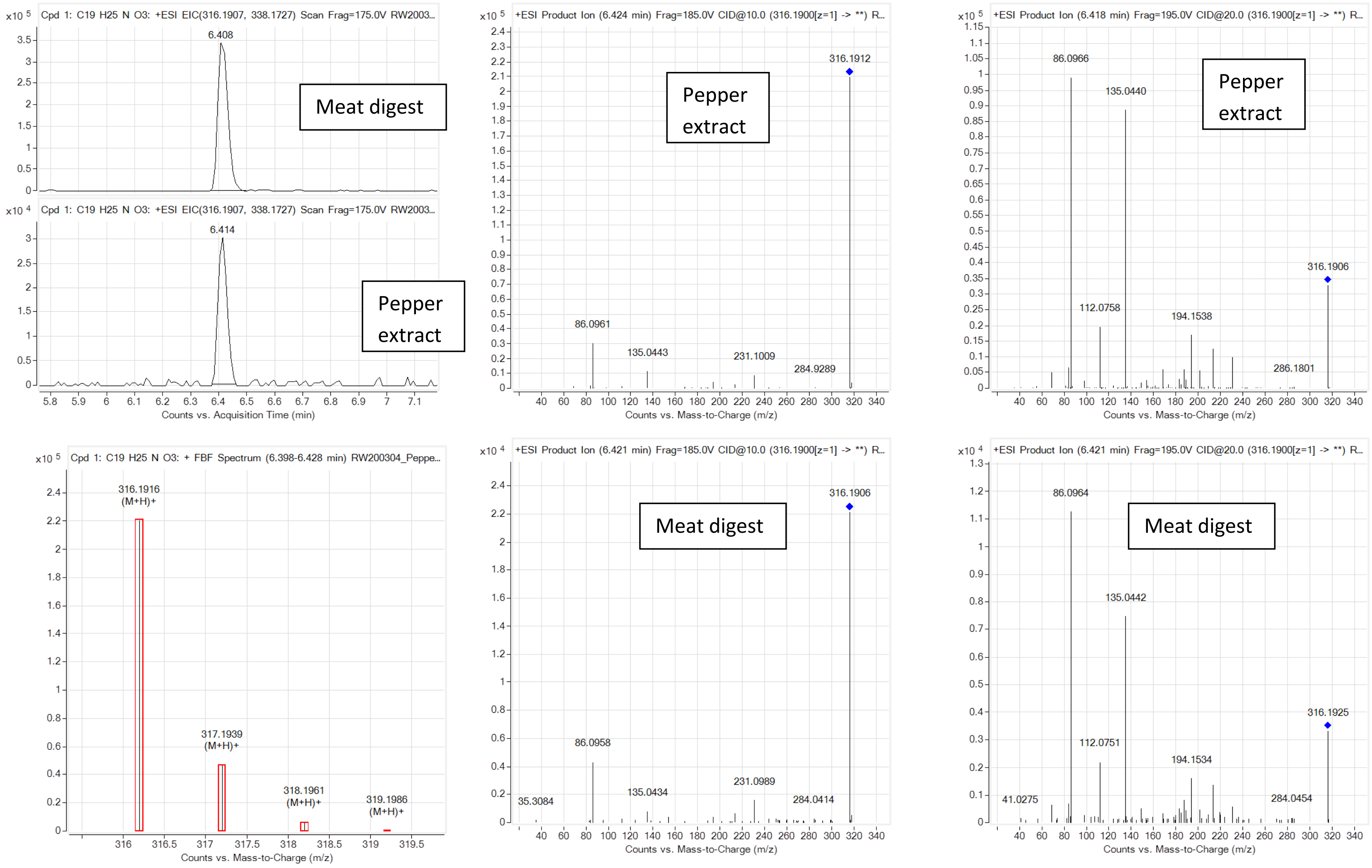

Online supporting material

FIGURE S8 : Annotation evidence for pepper compound 3 in fried sausage digest, comparison with compound in black pepper extract.

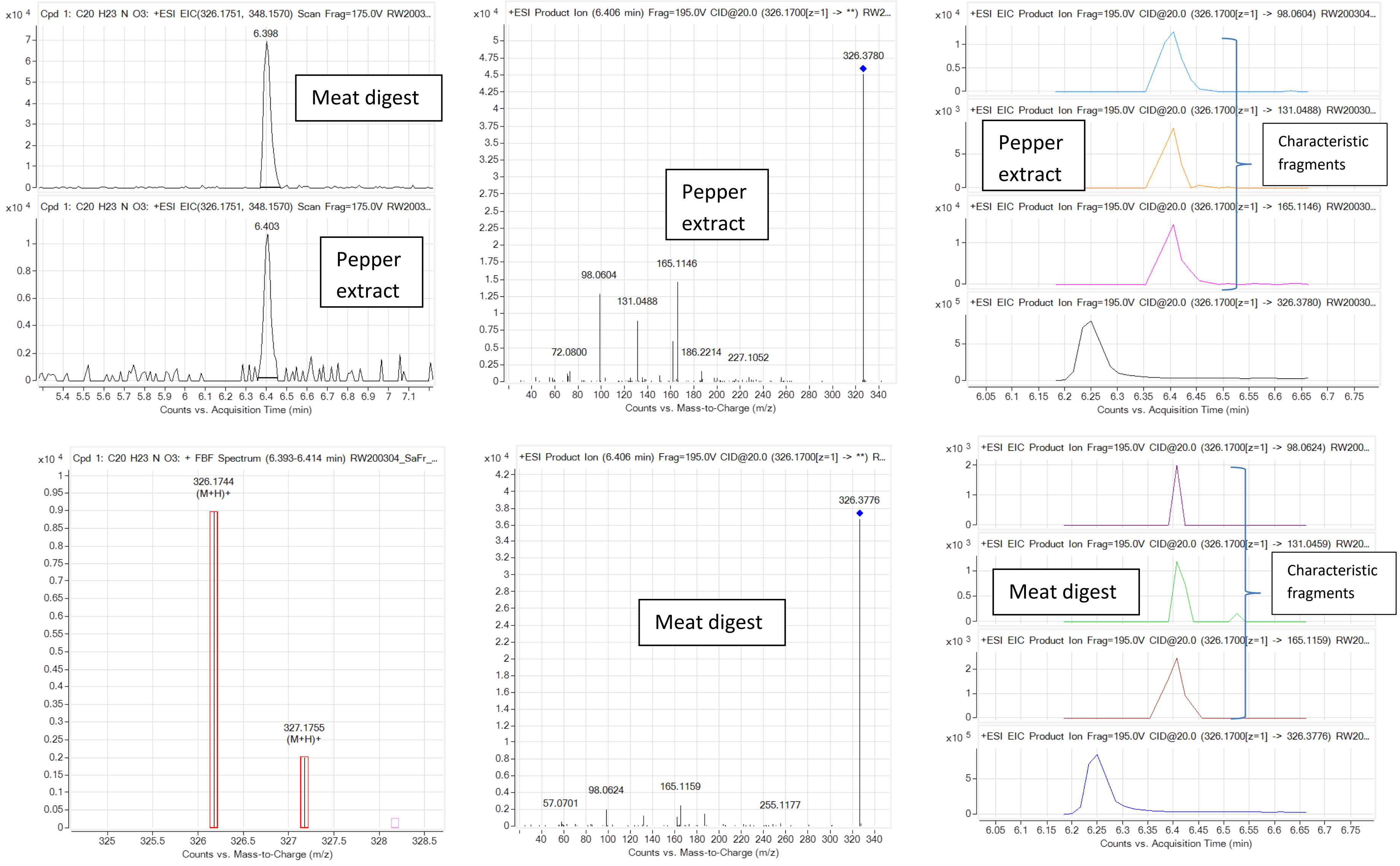

The same characteristic fragments (m/z = 98.06, m/z = 131.05, m/z = 165.12, RT = 6.4 min) are present in both pepper extract and meat digest, but a second compound has been selected for fragmentation (m/z = 326.38, RT = 6.25 min) which shows higher intensity and adds noise to the MS/MS spectrum.

FIGURE S9 : Annotation evidence for pepper compound 4 in fried sausage digest, comparison with compound in black pepper extract.

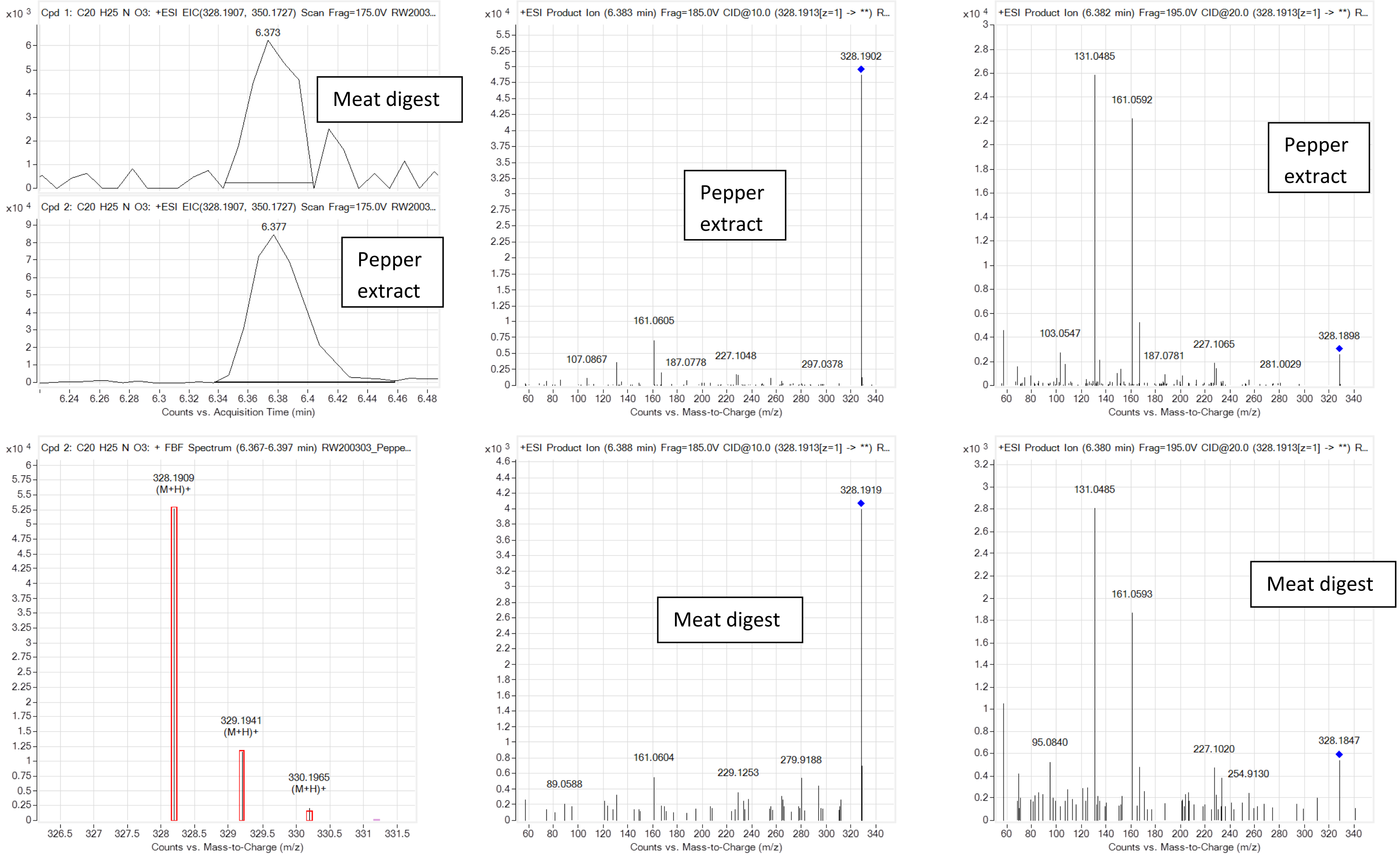

FIGURE S10 : Annotation evidence for pepper compound 5 in fried sausage digest, comparison with compound in black pepper extract.

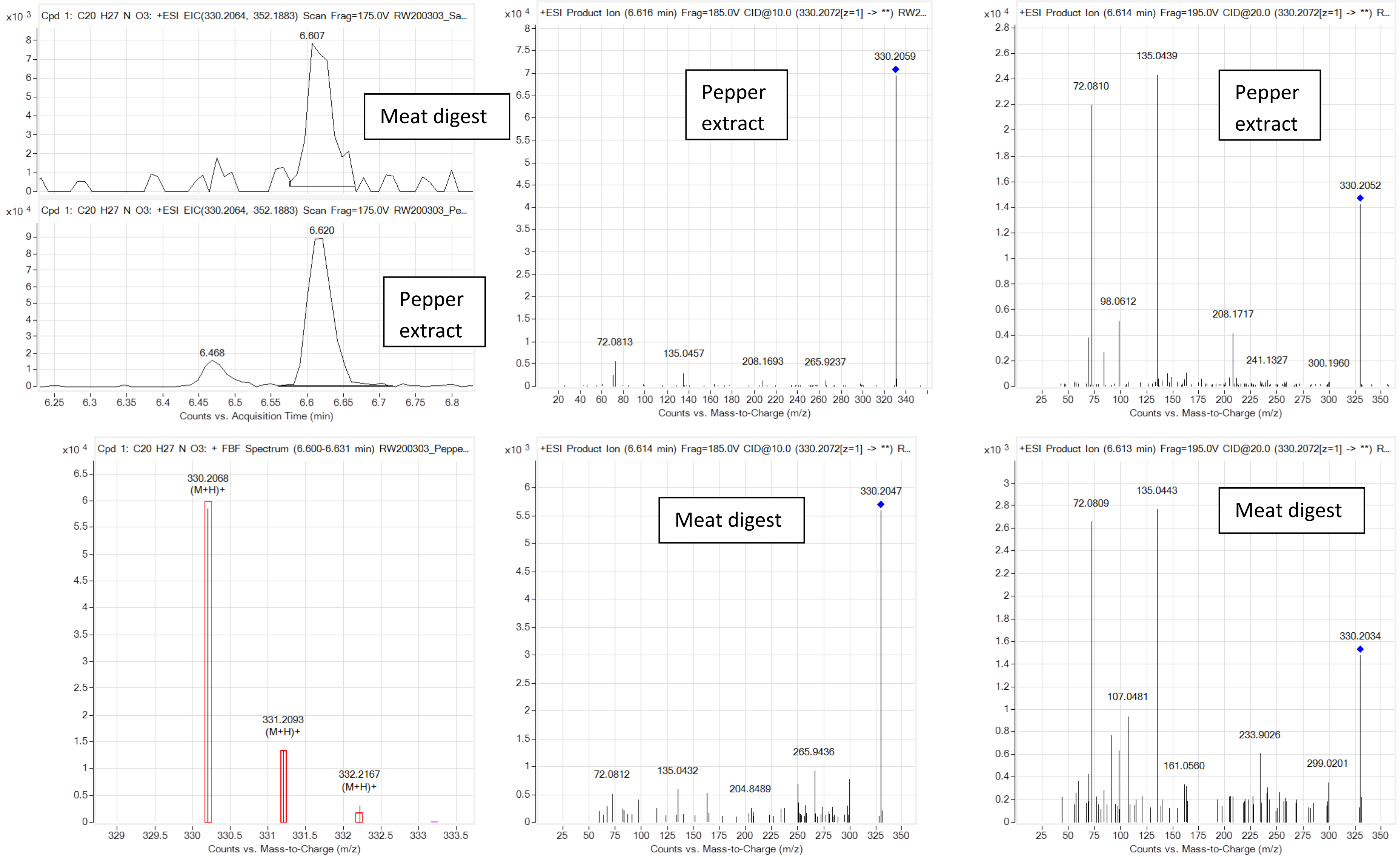

FIGURE S11 : Annotation evidence for pepper compound 6 in fried sausage digest, comparison with compound in black pepper extract.

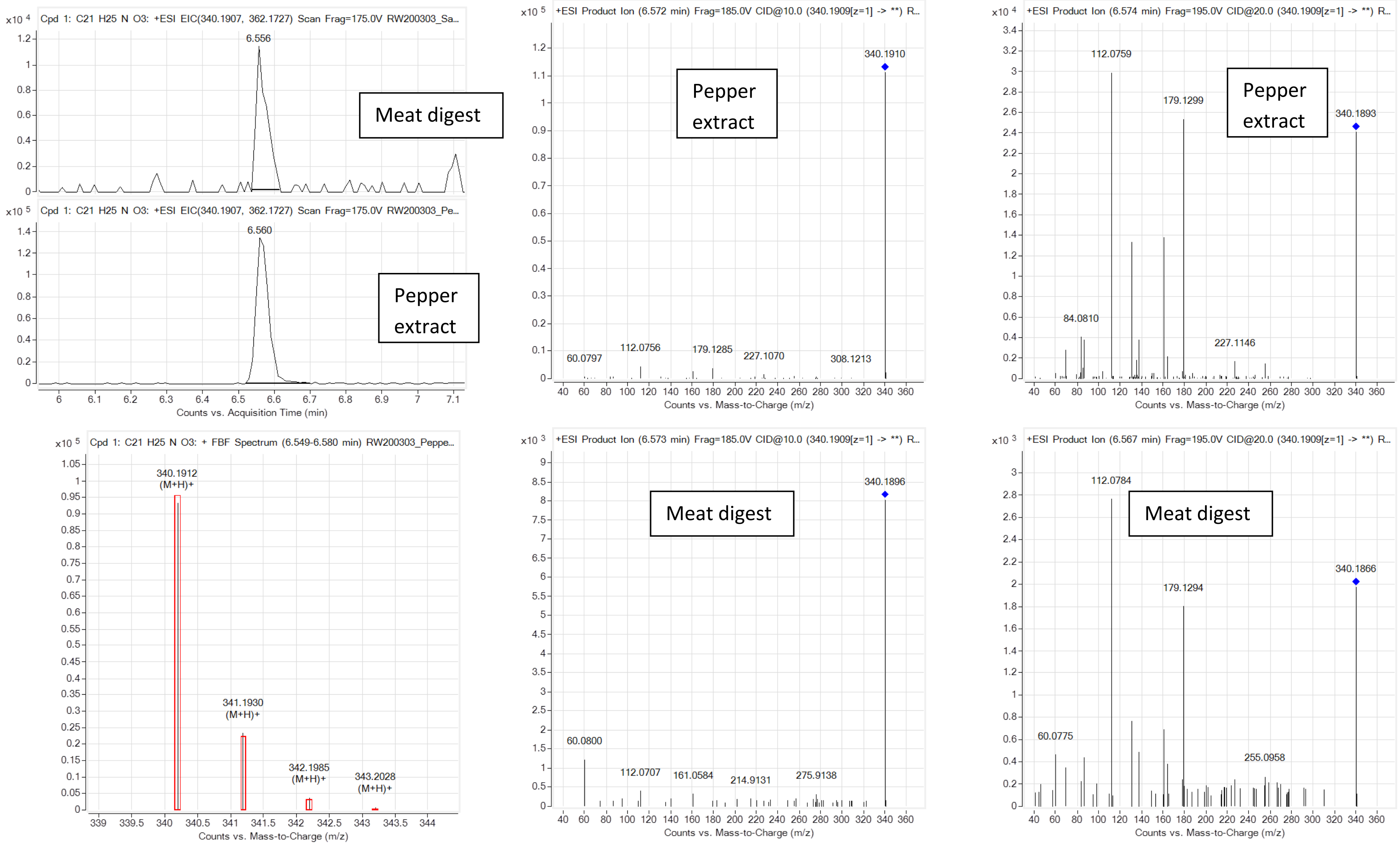

FIGURE S12 : Annotation evidence for pepper compound 7 in fried sausage digest, comparison with compound in black pepper extract.

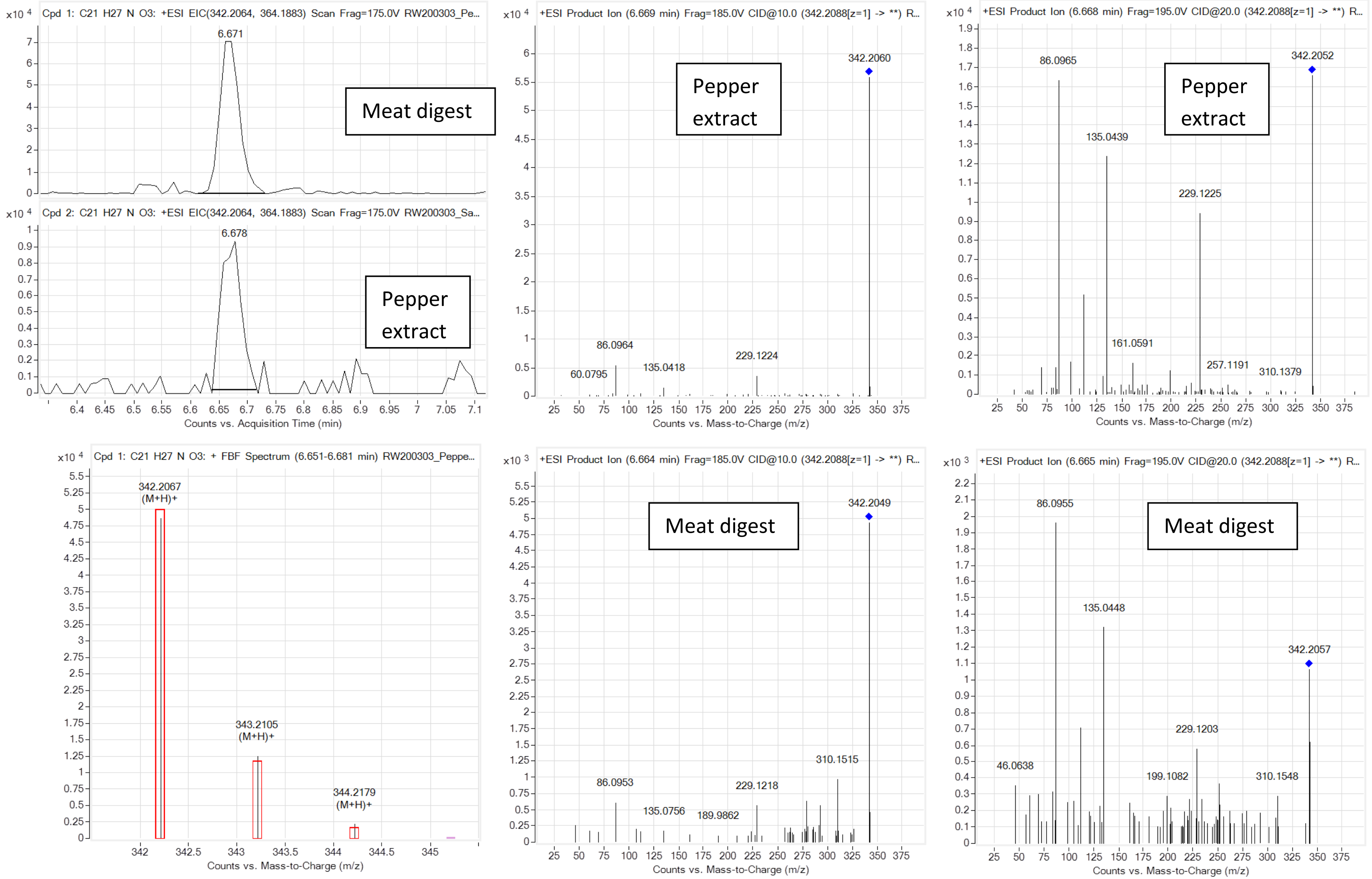

FIGURE S13 : Annotation evidence for pepper compound 8 in fried sausage digest, comparison with compound in black pepper extract.

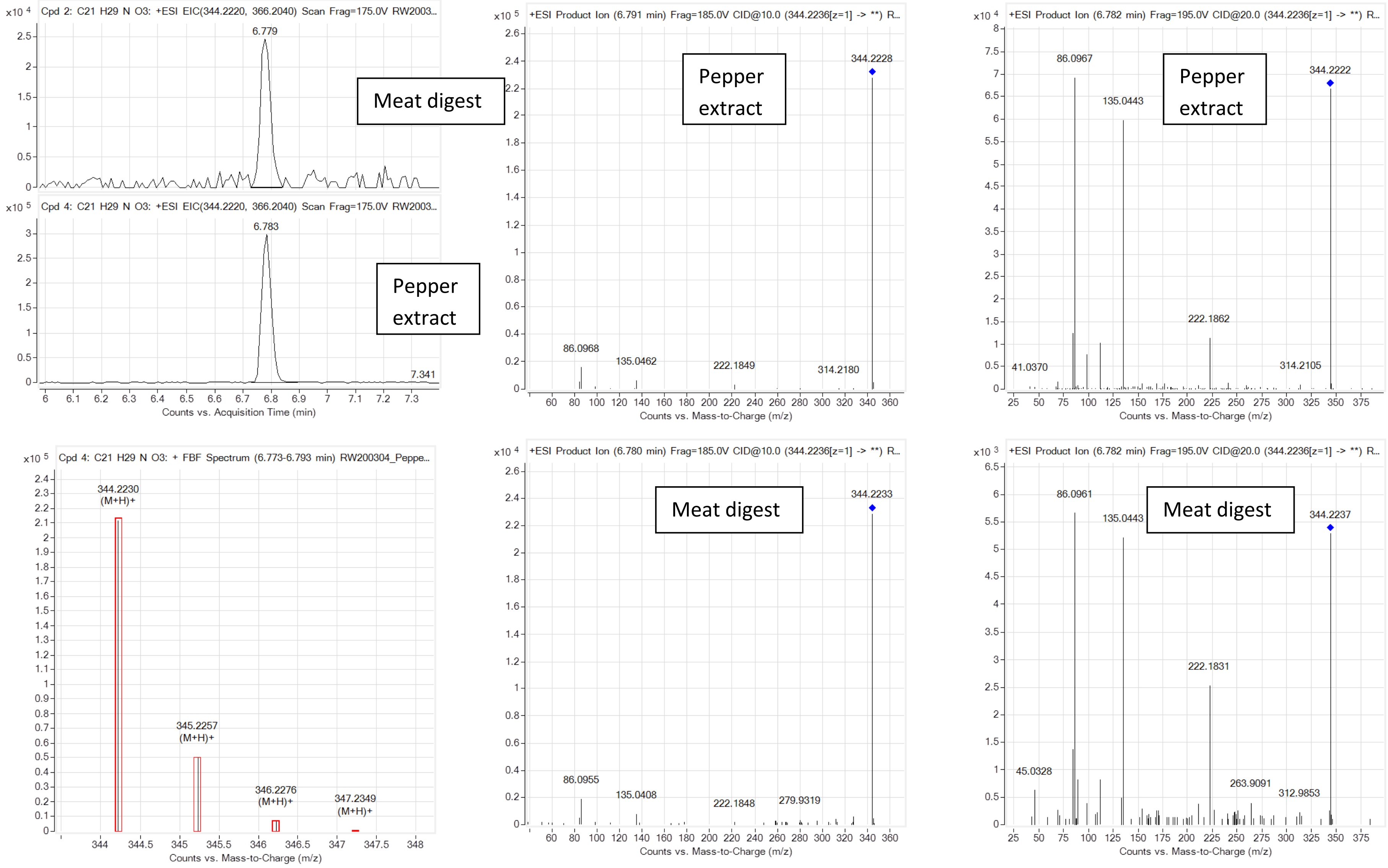

FIGURE S14 : Annotation evidence for pipercide in fried sausage digest, comparison with compound in black pepper extract.

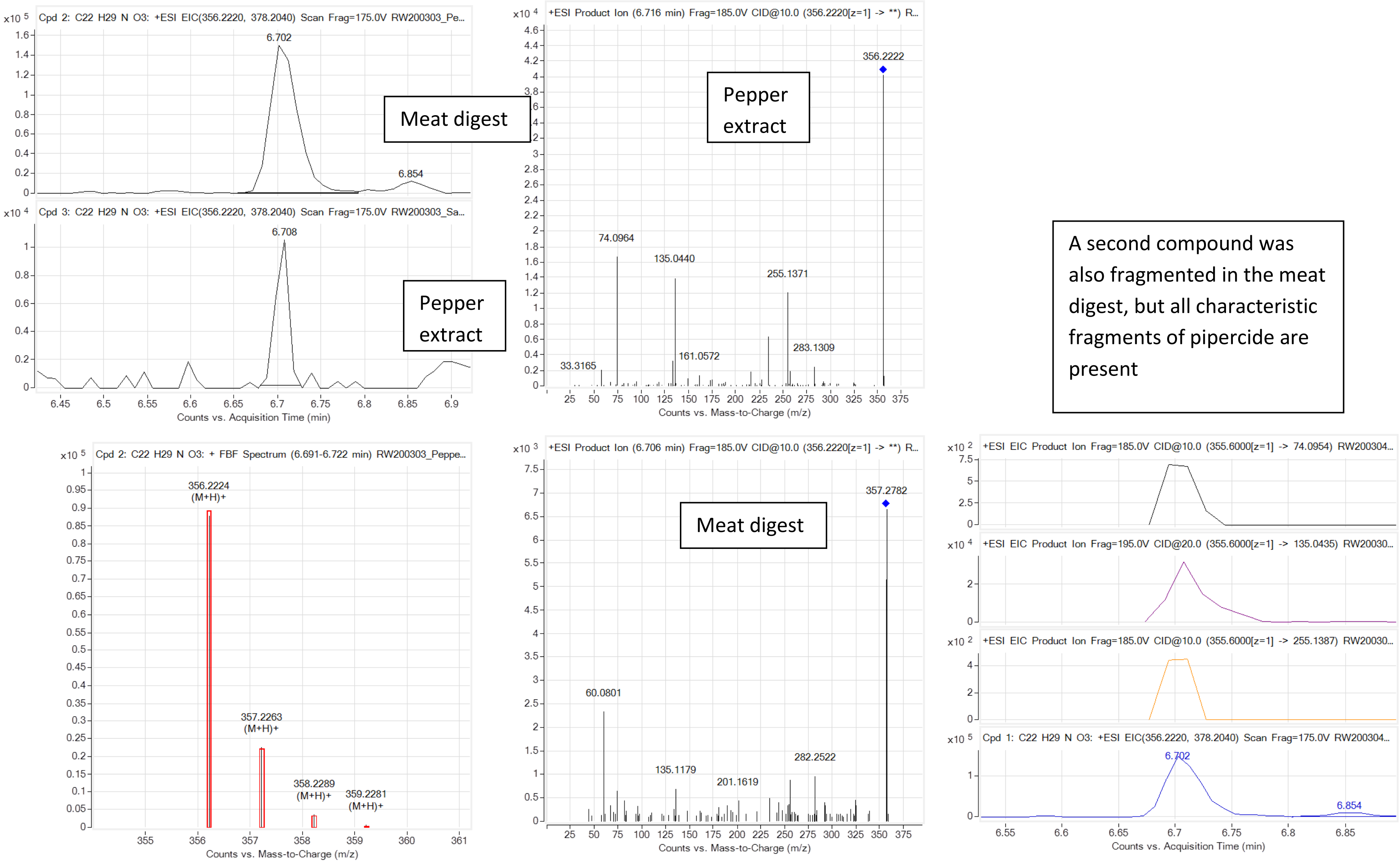

FIGURE S15 : Annotation evidence for phenylalanylisoleucine.

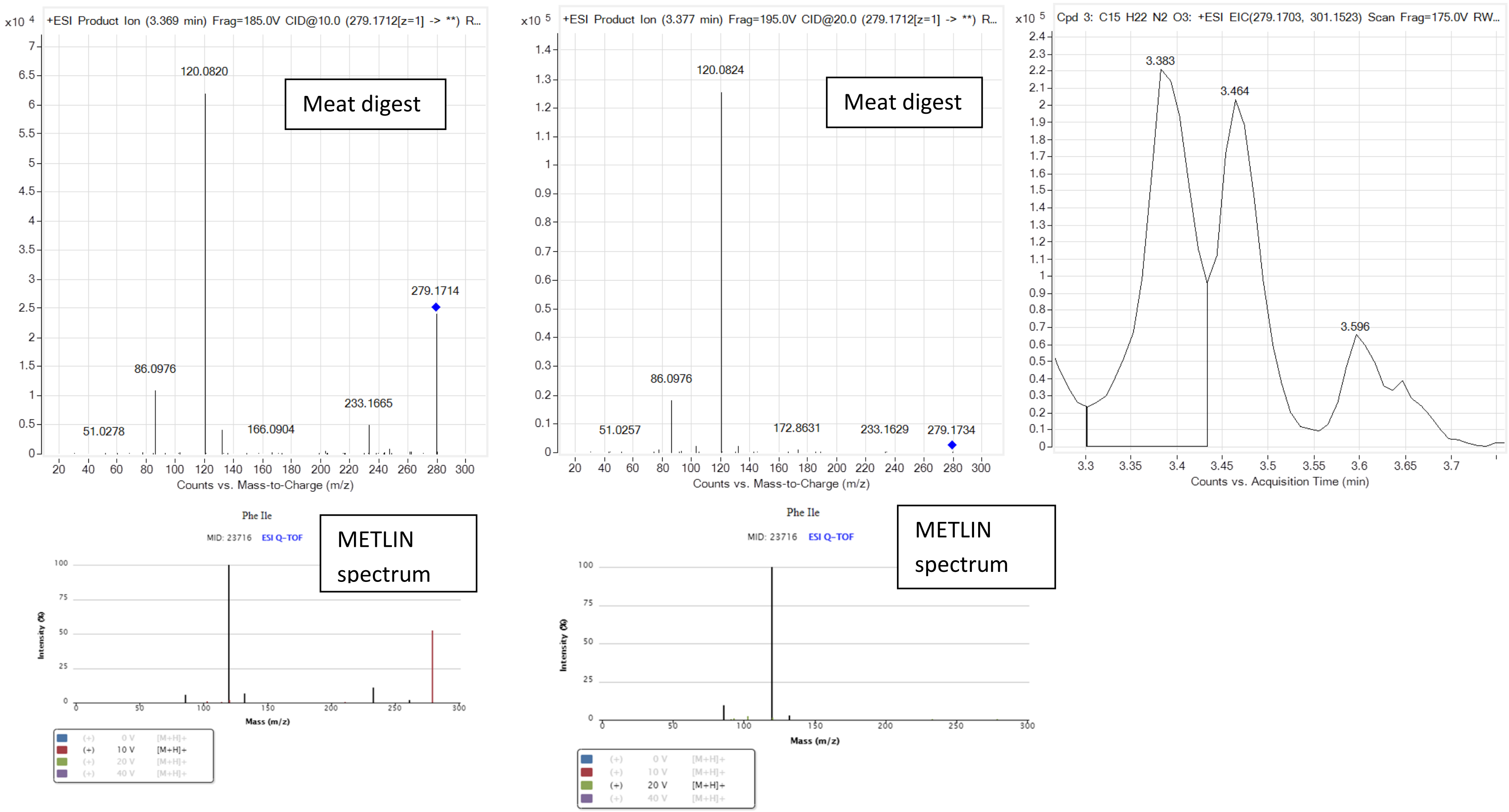

FIGURE S16 : Annotation evidence for γ-aminobutyric acid in fermented sausage digest, comparison with chemical standard.

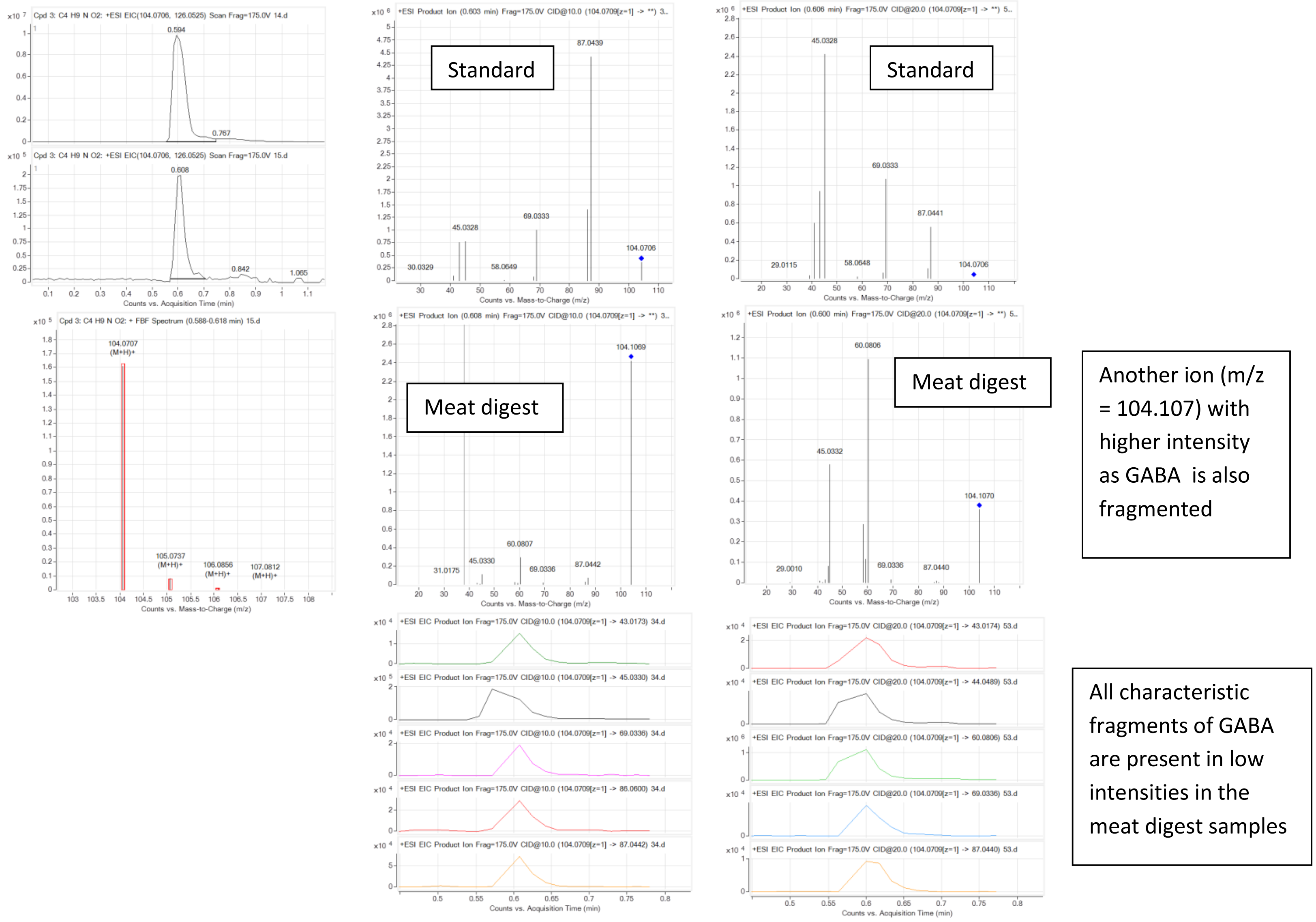

FIGURE S17 : Annotation evidence for histamine in fermented sausage digest, comparison with chemical standard.

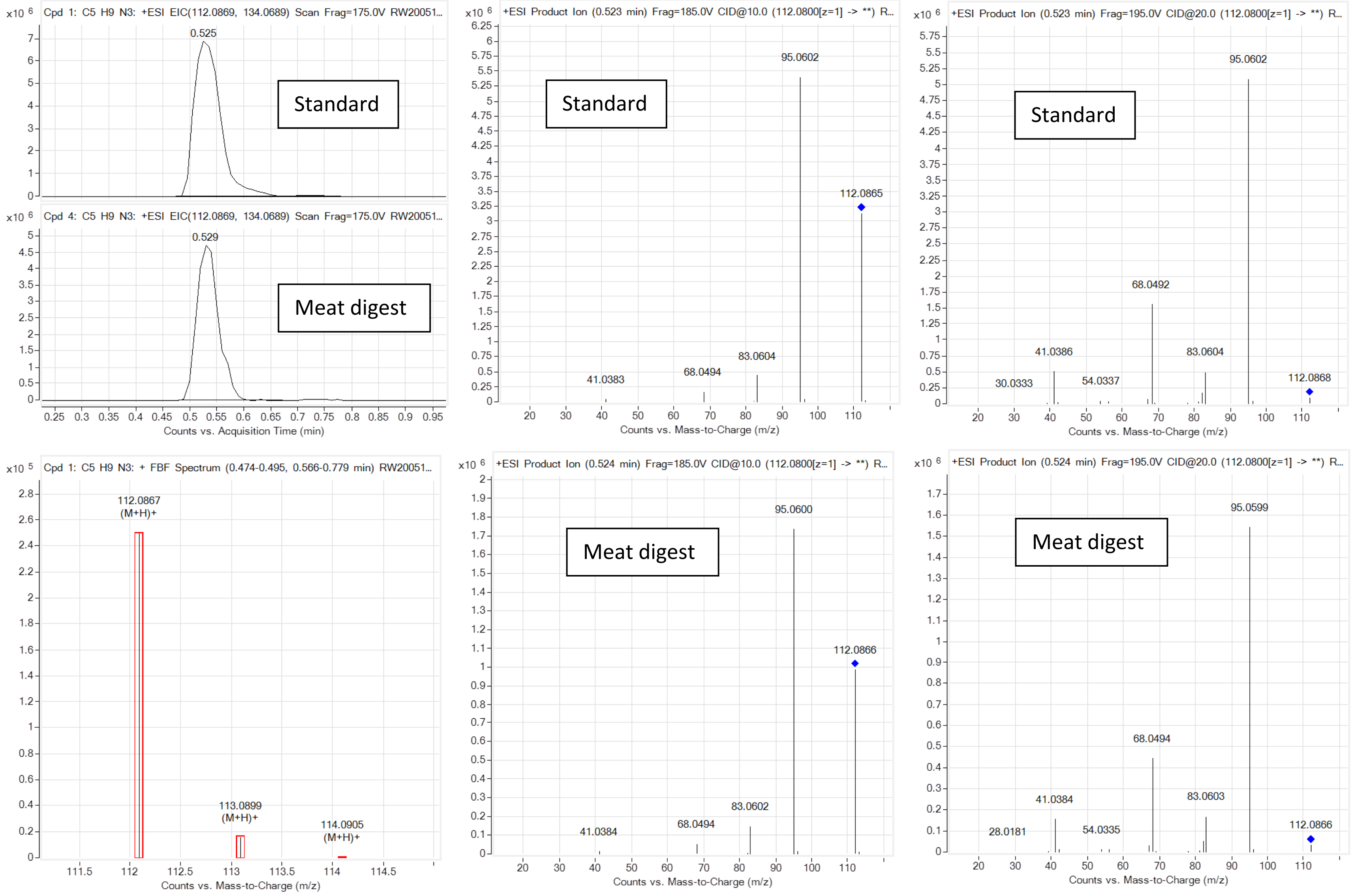

FIGURE S18: Annotation evidence for tyramine in fermented sausage digest, comparison with chemical standard.

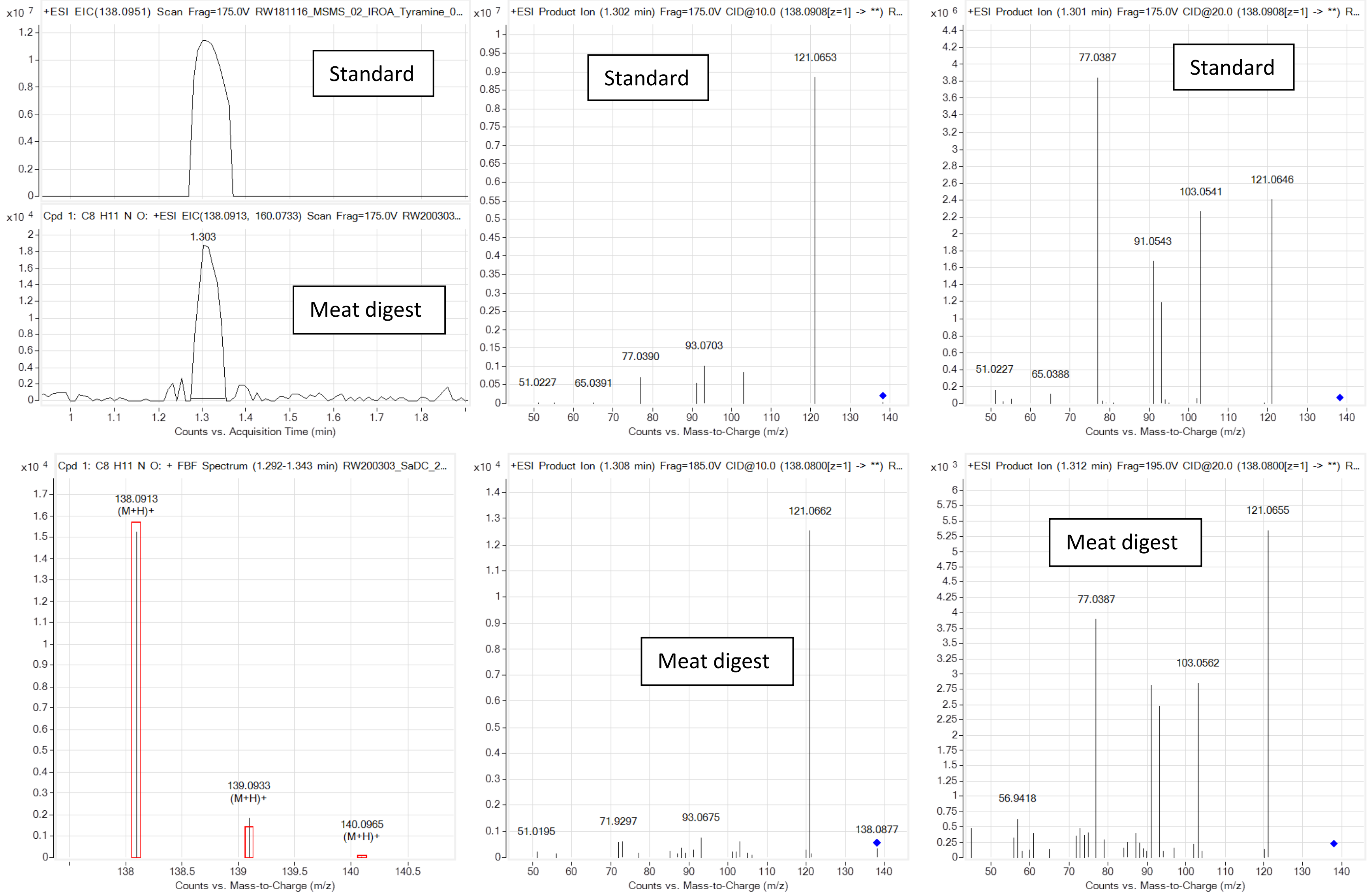

FIGURE S19 : Annotation evidence for tryptamine in fermented sausage digest, comparison with chemical standard.

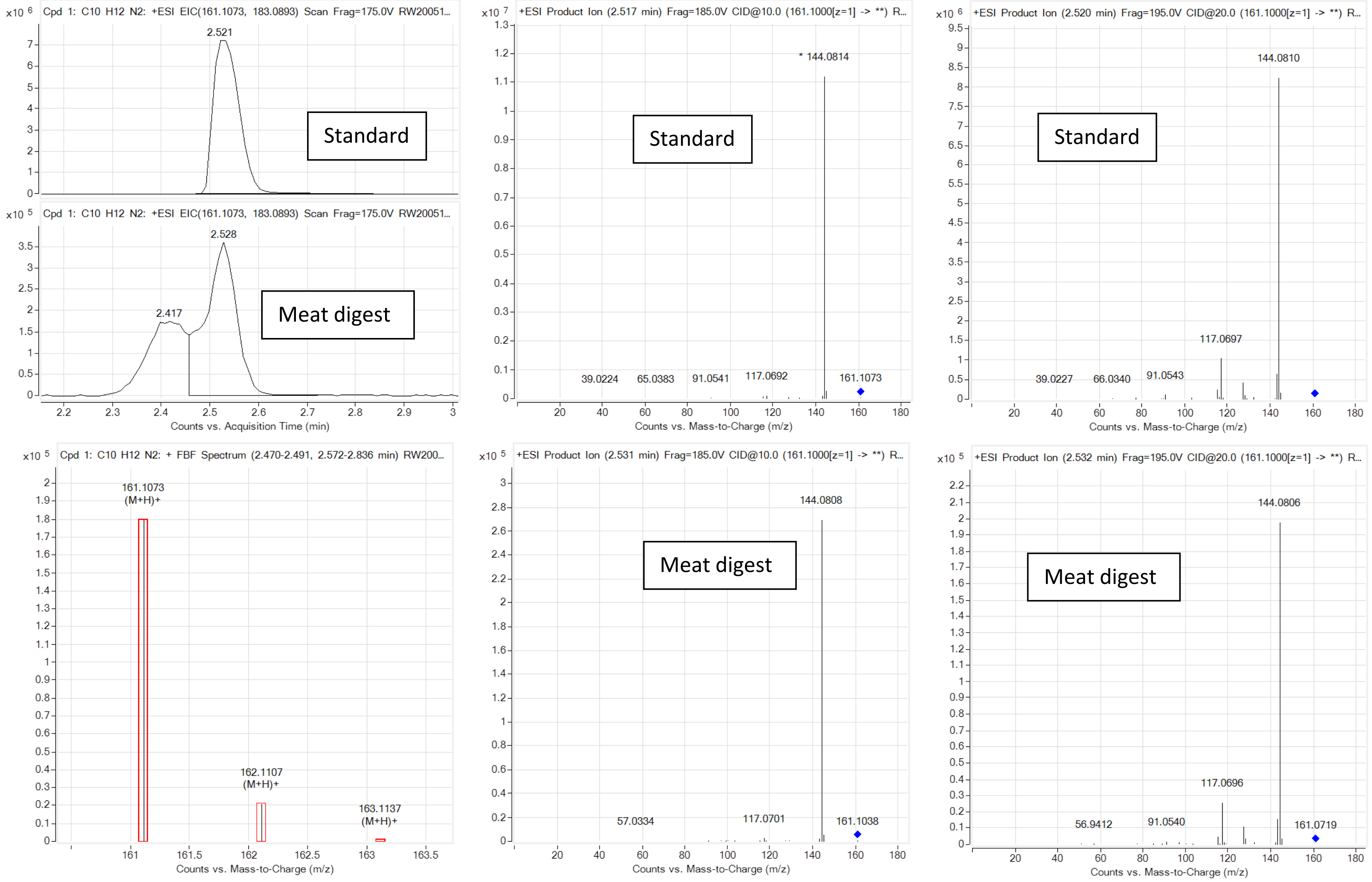

References:

1. Sumner, L.W.; Amberg, A.; Barrett, D.; Beale, M.H.; Beger, R.; Daykin, C.A.; Fan, T.W.-M.; Fiehn, O.; Goodacre, R.; Griffin, J.L.; et al. Proposed minimum reporting standards for chemical analysis Chemical Analysis Working Group (CAWG) Metabolomics Standards Initiative (MSI). *Metabolomics* **2007**, *3*, 211–221, doi:10.1007/s11306-007-0082-2.
2. Liu, H.-L.; Luo, R.; Chen, X.-Q.; Ba, Y.-Y.; Zheng, L.; Guo, W.-W.; Wu, X. Identification and simultaneous quantification of five alkaloids in Piper longum L. by HPLC-ESI-MS(n) and UFLC-ESI-MS/MS and their application to Piper nigrum L. *Food Chem* **2015**, *177*, 191–196, doi:10.1016/j.foodchem.2015.01.033.
3. Friedman, M.; Levin, C.E.; Lee, S.-U.; Lee, J.-S.; Ohnisi-Kameyama, M.; Kozukue, N. Analysis by HPLC and LC/MS of pungent piperamides in commercial black, white, green, and red whole and ground peppercorns. *J. Agric. Food Chem.* **2008**, *56*, 3028–3036, doi:10.1021/jf703711z.
4. Scott, I.M.; Puniani, E.; Jensen, H.; Livesey, J.F.; Poveda, L.; Sánchez-Vindas, P.; Durst, T.; Arnason, J.T. Analysis of Piperaceae Germplasm by HPLC and LCMS: A Method for Isolating and Identifying Unsaturated Amides from Piper spp Extracts. *J. Agric. Food Chem.* **2005**, *53*, 1907–1913, doi:10.1021/jf048305a.
5. Li, K.; Zhu, W.; Fu, Q.; Ke, Y.; Jin, Y.; Liang, X. Purification of amide alkaloids from Piper longum L. using preparative two-dimensional normal-phase liquid chromatography × reversed-phase liquid chromatography. *Analyst* **2013**, *138*, 3313–3320, doi:10.1039/C3AN00016H.
